# Supplementary material for: Selective knowledge sharing for privacy-preserving federated distillation without a good teacher
Source: Nat Commun. 2024 Jan 8;15:349. doi: 10.1038/s41467-023-44383-9 (PMC10774276; doi:10.1038/s41467-023-44383-9)
Supplement: Supplementary file 1 — Supplementary Information [file 41467_2023_44383_MOESM1_ESM.pdf]

Supplementary Information for “Selective Knowledge Sharing for Privacy-Preserving Federated Distillation without A Good Teacher”

Jiawei Shao[1], Fangzhao Wu[2,*], and Jun Zhang[1,*]

[1]Hong Kong University of Science and Technology, Hong Kong, China
[2]Microsoft Research Asia, Beijing, China
[*]correspondce: Jun Zhang (eejzhang@ust.hk), Fangzhao Wu (wufangzhao@gmail.com)

Supplementary Discussion

More Details of Experiments

In the experiments, we use stochastic gradient descent (SGD) as the optimizer. The learning rate is set to 0.1. The clients first train the local models independently based on their datasets for 200 SGD steps. In each communication round, the clients train the local models by using both local samples and proxy samples. In the non-IID settings, the clients perform 1-step and 10-step SGD training on the local datasets and the proxy dataset, respectively. In the IID setting, the clients perform 5-step and 6-step SGD training on the local datasets and the proxy dataset, respectively. To ensure a fair comparison, the clients in FedAvg perform 11-step SGD training per communication round. The architectures of local models are shown in Supplementary Table 1, Supplementary Table 2, and Supplementary Table 3. The convolutional layer with output channel o, kernel size k, and padding p is denoted as $\mathrm{Conv}(o,k,p)$. The fully-connected layer with output dimension o is defined as linear(o), and the max-pooling layer with kernel size k is denoted as MaxPool(k). ReLU represents the rectified linear unit function. On the CIFAR-10 dataset, our method estimates the density ratio of the extracted features to build the client-side selector. These features are the input of the fully-connected layer in ResNet. The ResNet models are pretrained on the ImageNet dataset.

We select four publicly available datasets to evaluate the performance of the proposed method. Supplementary Table 4 provides a summary of these datasets. In Selective-FD, each client adopts a portion of the local data as a validation set to determine the threshold τ_{client} of the client-side selector. In the weak non-IID scenario, the local datasets contain two classes. As directly modeling the joint distribution of these classes results in high sampling complexity, we construct two density-ratio estimators for these two classes, respectively. If any of the estimators indicates a proxy sample as an in-distribution sample, the corresponding local prediction will be used in knowledge distillation.

More Discussion on the Pneumonia Detection Task

In this part, we compare the local test accuracy of various methods on the pneumonia detection task. Specifically, we use local test sets to evaluate the performance of local models. We follow the same experimental setup as the main text except that we allocate 10% of the local private data for testing. The empirical results are shown in Supplementary Table 5. It is observed that all the models can perform well on the test datasets of Clients 3 and 4. This is because the local datasets of these two clients only contain one class of images. Besides, the FedED method has the worst performance since it trains only one global model through knowledge distillation, which cannot adapt to heterogeneous local datasets. Our Selective-FD method performs comparable or better than the baseline methods since it can effectively share knowledge among the clients. Moreover, compared with the results presented in the main text, the performance of IndepLearn is satisfactory. This is because the local models do not need to predict the labels of out-of-class images, and the classification task becomes easy.

More Discussion on Data Heterogenity

In the main text, we evaluated the performance of Selective-FD in the strong non-IID and weak non-IID settings, where each client only has one or two classes of samples. In this part, we consider more general non-IID settings by simulating the non-IID distribution based on the Dirichlet distribution $\mathrm{Dir}_K(\beta)$. The parameter K represents the number of clients, and $\beta > 0$ is a concentration parameter. When β is set to a smaller value, the data distribution is more non-IID. Specifically, we sample a vector $p_n \sim \mathrm{Dir}_K(\beta)$ and allocate a $p_{n,k}$ proportion of the instances of class n to client k. We conduct a performance comparison of the proposed Selective-FD method with two representative baselines, namely FedMD and IndepLearn, across various non-IID settings of the MNIST dataset. As shown in Supplementary Fig. 1, the accuracy of IndepLearn degrades with the decreasing of parameter β. This is because the data distribution becomes increasingly non-IID. The FedMD method demonstrates the ability to achieve good performance when $\beta > 10^{-1}$. However, it experiences performance degradation when $\beta < 10^{-1}$. In contrast, our Selective-FD method consistently maintains satisfactory performance, even when the parameter β decreases to 10^{-3}. Specifically, the accuracy gain of our method is more significant when using hard labels for knowledge distillation.

Ablation Study on Model Architecture

In this part, we extend the experiments to the transformer-based architecture to further verify the advantages of communication cost saving. Specifically, we compare the performance by adopting Multilayer Perceptron (MLP) and Vision in Transformers (ViT) as the backbone for the Fashion MNIST image classification task. The structure of MLP follows the setup in the main text. In the ViT model, the input patch size is 4, the depth of the encoder is 2, the number of heads is 8, and the output dimension per head is 512. The test accuracy and communication cost during the training process are shown in Supplementary Fig. 2. In line with the findings presented in the main text, our Selective-FD method exhibits lower accuracy compared to FedAvg while enhancing communication efficiency. Additionally, the ViT model incurs less communication overhead in comparison to the MLP model. This is because the fully-connected layers in an MLP have a substantial number of parameters.

More Discussion on Heterogeneous Computation Resources

We compare the training complexity of Selective-FD with FedAvg on the CIFAR-10 classification task. Consider that clients 1 to 4 utilize Jetson AGX Orin modules to train the models. Clients 5 to 8 and clients 9 to 10 own GPU servers equipped with the NVIDIA GeForce GTX 1080 Ti graphics card and NVIDIA GeForce RTX 2080 Ti graphics cards, respectively. Selective-FD allows clients to use different models for local training. A detailed summary of these model structures can be found in Supplementary Table 3. On the other hand, FedAvg initializes the global model as ResNet-50 in this experiment. The computation latency per round is shown in Supplementary Table 7. Compared with FedAvg, clients 1 to 4 in Selective-FD take half the computation time. This demonstrates the effectiveness of the model-agnostic FL method to reduce training complexity.

More Discussion on Proxy Data

For the sake of simplicity, our experiments utilize a part of the training data as the proxy samples to transfer knowledge. In real-world applications, the proxy data can be collected from publicly available datasets that are similar to the target application. The data platforms such as OpenDataLab, Paperswithcode, and HuggingFace provide extensive resources to accelerate the reuse of public datasets. Take the pneumonia detection task as an example. The proxy X-ray images can be produced from the public chest computerized tomography (CT) scan dataset. Another alternative method to tackle this problem is generating synthetic data for knowledge distillation. This involves training generators based on the local datasets and sharing generated samples among clients as proxy data.

Proof of Theorem 2

Prior to proving Theorem 2, we first present two Lemmas.

Lemma 1. *Given hypothesis spaces* $\mathscr{H} := \{\hat{\boldsymbol{h}} : \mathscr{X} \to V(\Delta^{C-1})\}$ *and* $\mathscr{G} := \{g : \mathscr{X} \to \{0,1\}\}$ *with* $g(\mathbf{x}) = \frac{1}{2}\|\hat{\boldsymbol{h}}(\mathbf{x}) - \hat{\boldsymbol{h}}'(\mathbf{x})\|_1$ *for* $\hat{\boldsymbol{h}}, \hat{\boldsymbol{h}}' \in \mathscr{H}$, *we have* $|\mathscr{L}_{\mathscr{D}}(\hat{\boldsymbol{h}}, \hat{\boldsymbol{h}}') - \mathscr{L}_{\mathscr{D}'}(\hat{\boldsymbol{h}}, \hat{\boldsymbol{h}}')| \le d_{\mathscr{G}}(\mathscr{D}, \mathscr{D}')$ *for* $\hat{\boldsymbol{h}}, \hat{\boldsymbol{h}}' \in \mathscr{H}$.

Proof.

$$d_{\mathscr{G}}(\mathscr{D}, \mathscr{D}') = 2 \sup_{g \in \mathscr{G}} \left| \Pr_{\mathscr{D}}[g(\mathbf{x}) = 1] - \Pr_{\mathscr{D}'}[g(\mathbf{x}) = 1] \right|, \tag{1}$$

$$= \sup_{\hat{\boldsymbol{h}}, \hat{\boldsymbol{h}}' \in \mathscr{H}} 2 \left| \frac{1}{2}\mathbb{E}_{\mathbf{x}\sim\mathscr{D}}[\hat{\boldsymbol{h}}(\mathbf{x}) - \hat{\boldsymbol{h}}'(\mathbf{x})] - \frac{1}{2}\mathbb{E}_{\mathbf{x}\sim\mathscr{D}'}[\hat{\boldsymbol{h}}(\mathbf{x}) - \hat{\boldsymbol{h}}'(\mathbf{x})] \right| \ge |\mathscr{L}_{\mathscr{D}}(\hat{\boldsymbol{h}}, \hat{\boldsymbol{h}}') - \mathscr{L}_{\mathscr{D}'}(\hat{\boldsymbol{h}}, \hat{\boldsymbol{h}}')|. \tag{2}$$

Lemma 2. *For any* $\delta \in (0,1)$, *with probability at least* $1-\delta$ *over the choice of the samples, we have*

$$\mathscr{L}_{\mathscr{D}_{\text{test}}}(\hat{\boldsymbol{h}}, \hat{\boldsymbol{h}}^*) \le \mathscr{L}_{\hat{\mathscr{D}}_k \cup \hat{\mathscr{D}}_{\text{proxy}}}(\hat{\boldsymbol{h}}) + \sqrt{\left(\frac{2\alpha^2}{m_k} + \frac{2(1-\alpha)^2}{m_{\text{proxy}}}\right) \log \frac{2}{\delta}}. \tag{3}$$

Proof. The loss $\mathscr{L}_{\hat{\mathscr{D}}_k \cup \hat{\mathscr{D}}_{\text{proxy}}}(\hat{\boldsymbol{h}})$ can be written as

$$\mathscr{L}_{\hat{\mathscr{D}}_k \cup \hat{\mathscr{D}}_{\text{proxy}}}(\hat{\boldsymbol{h}}) = \alpha \mathscr{L}_{\hat{\mathscr{D}}_k}(\hat{\boldsymbol{h}}) + (1-\alpha)\mathscr{L}_{\hat{\mathscr{D}}_{\text{proxy}}}(\hat{\boldsymbol{h}}), \tag{4}$$

$$= \frac{1}{m_k + m_{\text{proxy}}} \left[\sum_{\mathbf{x} \in \hat{\mathscr{D}}_k} \frac{\alpha(m_k + m_{\text{proxy}})}{m_k} \|\hat{\boldsymbol{h}}(\mathbf{x}) - \hat{\boldsymbol{h}}^*(\mathbf{x})\|_1 + \sum_{\mathbf{x} \in \hat{\mathscr{D}}_{\text{proxy}}} \frac{(1-\alpha)(m_k + m_{\text{proxy}})}{m_{\text{proxy}}} \|\hat{\boldsymbol{h}}(\mathbf{x}) - \hat{\boldsymbol{h}}^*(\mathbf{x})\|_1 \right]. \tag{5}$$

Let $X_1^{(k)}, \ldots, X_{m_k}^{(k)}$ and $X_1^{(\text{proxy})}, \ldots, X_{m_{\text{proxy}}}^{(\text{proxy})}$ be independent random variables that take on the values of $\frac{\alpha(m_k+m_{\text{proxy}})}{m_k}\|\hat{\boldsymbol{h}}(\mathbf{x}) - \hat{\boldsymbol{h}}^*(\mathbf{x})\|_1$ for $\mathbf{x} \in \hat{\mathscr{D}}_k$ and $\frac{(1-\alpha)(m_k+m_{\text{proxy}})}{m_{\text{proxy}}}\|\hat{\boldsymbol{h}}(\mathbf{x}) - \hat{\boldsymbol{h}}^*(\mathbf{x})\|_1$ for $\mathbf{x} \in \hat{\mathscr{D}}_{\text{proxy}}$, respectively. We define $\overline{X}$ as the mean value of these variables, which represents the empirical loss in (5). By linearity of expectations, $\mathbb{E}[\overline{X}]$ is equal to the loss $\mathscr{L}_{\mathscr{D}_k \cup \mathscr{D}_{\text{proxy}}}(\hat{\boldsymbol{h}})$. According to Hoeffding's inequality, for any $\varepsilon > 0$, we have

$$\Pr\left[|\mathscr{L}_{\mathscr{D}_k \cup \mathscr{D}_{\text{proxy}}}(\hat{\boldsymbol{h}}) - \mathscr{L}_{\hat{\mathscr{D}}_k \cup \hat{\mathscr{D}}_{\text{proxy}}}(\hat{\boldsymbol{h}})| \ge \varepsilon \right] \le 2\exp\left(\frac{-\varepsilon^2}{\frac{2\alpha^2}{m_k} + \frac{2(1-\alpha)^2}{m_{\text{proxy}}}} \right). \tag{6}$$

Let the right-hand side of (6) be δ. We can derive the inequality in Theorem 2.

Denote $\hat{\boldsymbol{h}}_k^* = \arg\min_{\hat{\boldsymbol{h}}_k \in \mathcal{H}_k} \left\{ \mathcal{L}_{\mathcal{D}_{\text{test}}}(\hat{\boldsymbol{h}}_k, \hat{\boldsymbol{h}}^*) + \mathcal{L}_{\mathcal{D}_k}(\hat{\boldsymbol{h}}_k, \hat{\boldsymbol{h}}^*) \right\}$ and $\hat{\boldsymbol{h}}_{k,\text{proxy}}^* = \arg\min_{\hat{\boldsymbol{h}}_k \in \mathcal{H}_k} \left\{ \mathcal{L}_{\mathcal{D}_{\text{test}}}(\hat{\boldsymbol{h}}_k, \hat{\boldsymbol{h}}^*) + \mathcal{L}_{\mathcal{D}_{\text{proxy}}}(\hat{\boldsymbol{h}}_k, \hat{\boldsymbol{h}}^*) \right\}$. We are now ready to prove Theorem 2.

Proof. The following derives the upper bound of $|\mathcal{L}_{\mathcal{D}_{\text{test}}}(\hat{\boldsymbol{h}}, \hat{\boldsymbol{h}}^*) - \mathcal{L}_{\mathcal{D}_k \cup \mathcal{D}_{\text{proxy}}}(\hat{\boldsymbol{h}})|$:

$$
\begin{aligned}
&|\mathcal{L}_{\mathcal{D}_{\text{test}}}(\hat{\boldsymbol{h}}, \hat{\boldsymbol{h}}^*) - \mathcal{L}_{\mathcal{D}_k \cup \mathcal{D}_{\text{proxy}}}(\hat{\boldsymbol{h}})| = |\mathcal{L}_{\mathcal{D}_{\text{test}}}(\hat{\boldsymbol{h}}, \hat{\boldsymbol{h}}^*) - \alpha \mathcal{L}_{\mathcal{D}_k}(\hat{\boldsymbol{h}}, \hat{\boldsymbol{h}}^*) - (1-\alpha)\mathcal{L}_{\mathcal{D}_{\text{proxy}}}(\hat{\boldsymbol{h}}, \boldsymbol{h}_{\text{proxy}}^*)|, && (7)\\
\le& \alpha |\mathcal{L}_{\mathcal{D}_{\text{test}}}(\hat{\boldsymbol{h}}, \hat{\boldsymbol{h}}^*) - \mathcal{L}_{\mathcal{D}_k}(\hat{\boldsymbol{h}}, \hat{\boldsymbol{h}}^*)| + (1-\alpha)|\mathcal{L}_{\mathcal{D}_{\text{test}}}(\hat{\boldsymbol{h}}, \hat{\boldsymbol{h}}^*) - \mathcal{L}_{\mathcal{D}_{\text{proxy}}}(\hat{\boldsymbol{h}}, \boldsymbol{h}_{\text{proxy}}^*)|, && (8)\\
\le& \alpha \Big[|\mathcal{L}_{\mathcal{D}_k}(\hat{\boldsymbol{h}}, \hat{\boldsymbol{h}}^*) - \mathcal{L}_{\mathcal{D}_k}(\hat{\boldsymbol{h}}, \hat{\boldsymbol{h}}_k^*)| + |\mathcal{L}_{\mathcal{D}_k}(\hat{\boldsymbol{h}}, \hat{\boldsymbol{h}}_k^*) - \mathcal{L}_{\mathcal{D}_{\text{test}}}(\hat{\boldsymbol{h}}, \hat{\boldsymbol{h}}_k^*)| + |\mathcal{L}_{\mathcal{D}_{\text{test}}}(\hat{\boldsymbol{h}}, \hat{\boldsymbol{h}}_k^*) - \mathcal{L}_{\mathcal{D}_{\text{test}}}(\hat{\boldsymbol{h}}, \hat{\boldsymbol{h}}^*)| \Big], && (9)\\
&+ (1-\alpha) \Big[|\mathcal{L}_{\mathcal{D}_{\text{proxy}}}(\hat{\boldsymbol{h}}, \boldsymbol{h}_{\text{proxy}}^*) - \mathcal{L}_{\mathcal{D}_{\text{proxy}}}(\hat{\boldsymbol{h}}, \hat{\boldsymbol{h}}_{k,\text{proxy}}^*)| + |\mathcal{L}_{\mathcal{D}_{\text{proxy}}}(\hat{\boldsymbol{h}}, \hat{\boldsymbol{h}}_{k,\text{proxy}}^*) - \mathcal{L}_{\mathcal{D}_{\text{test}}}(\hat{\boldsymbol{h}}, \hat{\boldsymbol{h}}_{k,\text{proxy}}^*)| && (10)\\
&+ |\mathcal{L}_{\mathcal{D}_{\text{test}}}(\hat{\boldsymbol{h}}, \hat{\boldsymbol{h}}_{k,\text{proxy}}^*) - \mathcal{L}_{\mathcal{D}_{\text{test}}}(\hat{\boldsymbol{h}}, \hat{\boldsymbol{h}}^*)| \Big], && (11)\\
\le& \alpha \Big[\mathcal{L}_{\mathcal{D}_k}(\hat{\boldsymbol{h}}_k^*, \hat{\boldsymbol{h}}^*) + |\mathcal{L}_{\mathcal{D}_k}(\hat{\boldsymbol{h}}, \hat{\boldsymbol{h}}_k^*) - \mathcal{L}_{\mathcal{D}_{\text{test}}}(\hat{\boldsymbol{h}}, \hat{\boldsymbol{h}}_k^*)| + \mathcal{L}_{\mathcal{D}_{\text{test}}}(\hat{\boldsymbol{h}}_k^*, \hat{\boldsymbol{h}}^*) \Big], && (12)\\
&+ (1-\alpha) \Big[\mathcal{L}_{\mathcal{D}_{\text{proxy}}}(\hat{\boldsymbol{h}}_{k,\text{proxy}}^*, \boldsymbol{h}_{\text{proxy}}^*) + |\mathcal{L}_{\mathcal{D}_{\text{proxy}}}(\hat{\boldsymbol{h}}, \hat{\boldsymbol{h}}_{k,\text{proxy}}^*) - \mathcal{L}_{\mathcal{D}_{\text{test}}}(\hat{\boldsymbol{h}}, \hat{\boldsymbol{h}}_{k,\text{proxy}}^*)| + \mathcal{L}_{\mathcal{D}_{\text{test}}}(\hat{\boldsymbol{h}}_{k,\text{proxy}}^*, \hat{\boldsymbol{h}}^*) \Big], && (13)\\
\le& \alpha \left[\lambda_k + d_{\mathcal{G}_k}(\mathcal{D}_k, \mathcal{D}_{\text{test}}) \right] + (1-\alpha) \Big[\lambda_{k,\text{proxy}} + d_{\mathcal{G}_k}(\mathcal{D}_{\text{proxy}}, \mathcal{D}_{\text{test}}) + \mathcal{L}_{\mathcal{D}_{\text{proxy}}}(\hat{\boldsymbol{h}}_{k,\text{proxy}}^*, \boldsymbol{h}_{\text{proxy}}^*) - \mathcal{L}_{\mathcal{D}_{\text{proxy}}}(\hat{\boldsymbol{h}}_{k,\text{proxy}}^*, \hat{\boldsymbol{h}}^*) \Big], && (14)\\
\le& \alpha \left[\lambda_k + d_{\mathcal{G}_k}(\mathcal{D}_k, \mathcal{D}_{\text{test}}) \right] + (1-\alpha) \Big[\lambda_{k,\text{proxy}} + d_{\mathcal{G}_k}(\mathcal{D}_{\text{proxy}}, \mathcal{D}_{\text{test}}) + \mathcal{L}_{\mathcal{D}_{\text{proxy}}}(\boldsymbol{h}_{\text{proxy}}^*, \hat{\boldsymbol{h}}^*) \Big], && (15)
\end{aligned}
$$

where

$$
\mathcal{L}_{\mathcal{D}_{\text{proxy}}}(\boldsymbol{h}_{\text{proxy}}^*, \hat{\boldsymbol{h}}^*) = p_{\text{proxy}}^{(1)} \mathcal{L}_{\mathcal{D}_{\text{proxy}}^{(1)}}(\hat{\boldsymbol{h}}^*, \boldsymbol{h}_{\text{proxy}}^*) + p_{\text{proxy}}^{(2)} \mathcal{L}_{\mathcal{D}_{\text{proxy}}^{(2)}}(\hat{\boldsymbol{h}}_{\text{proxy}}^*, \boldsymbol{h}_{\text{proxy}}^*). \tag{16}
$$

The triangle inequality gives rise to (12), (13), and (15), while Lemma 1 underlies the inequality (14). By combining (15), (16), and Lemma 2, we obtain the upper bound stated in Theorem 2.

Ablation Study on α in Theorem 2

In this part, we investigate the effect of coefficient α on the error bound in Theorem 2. We first identify the negligible terms. Consider the deep learning model at client k has enough parameters such that its hypothesis space $\mathcal{H}_k$ contains the ground truth labeling function $\hat{\boldsymbol{h}}^*$. In this case, both λ_k and $\lambda_{k,\text{proxy}}$ hold a value of zero. Besides, the numerical constraint tends to be zero given sufficient training samples. Furthermore, the proxy dataset $\mathcal{D}_{\text{proxy}}$ for knowledge distillation is expected to be less heterogeneous compared with the local heterogeneous dataset $\mathcal{D}_k$ at client k. Thus, the distance $\mathcal{G}_k(\mathcal{D}_{\text{proxy}}, \mathcal{D}_{\text{test}})$ is much smaller than $\mathcal{G}_k(\mathcal{D}_k, \mathcal{D}_{\text{test}})$. If the proxy dataset follows the same distribution as the test set, the $\mathcal{G}_k(\mathcal{D}_{\text{proxy}}, \mathcal{D}_{\text{test}})$ distance equals to zero.

Now it is clear that when α approaches 1, the empirical risk and the distance $\mathcal{G}_k(\mathcal{D}_k, \mathcal{D}_{\text{test}})$ dominate the error bound. When α is close to 0, the empirical risk, misleading knowledge, and ambiguous knowledge become the dominant factors. To support this analysis, we conduct an ablation study on MNIST under a weak non-IID setting, comparing the classification error across various α values. Our method utilizes the proposed selection mechanism as a pseudo-labeling function of the proxy dataset $\boldsymbol{h}_{\text{proxy}}^*$. The local client k trains its local model by minimizing the empirical risk $\alpha \mathcal{L}_{\hat{\mathcal{D}}_k}(\hat{\boldsymbol{h}}_k, \hat{\boldsymbol{h}}^*) + (1-\alpha)\mathcal{L}_{\hat{\mathcal{D}}_{\text{proxy}}}(\hat{\boldsymbol{h}}_k, \boldsymbol{h}_{\text{proxy}}^*)$. To better assess the negative impact of misleading and ambiguous knowledge, we consider a baseline where the proxy dataset has ground truth labels $\hat{\boldsymbol{h}}^*(\mathbf{x})$. The client minimizes the combined loss $\alpha \mathcal{L}_{\hat{\mathcal{D}}_k}(\hat{\boldsymbol{h}}_k, \hat{\boldsymbol{h}}^*) + (1-\alpha)\mathcal{L}_{\hat{\mathcal{D}}_{\text{proxy}}}(\hat{\boldsymbol{h}}_k, \hat{\boldsymbol{h}}^*)$ to train the local model.

As shown in Supplementary Fig. 3, the test error rate of the baseline decreases monotonously as the α value decreases. This is because a small α reduces the negative influence of the local heterogeneous dataset $\mathcal{D}_k$ on the training process. In contrast, the error rate of our proposed method first decreases but then increases when α approaches 0. Notably, this error rate is consistently higher compared to that of the baseline. These results can primarily be attributed to misleading and ambiguous knowledge, which degrades the training performance, particularly when α is close to 0.

Supplementary Tables

Client 1	Client 2	Client 3	Client 4
Conv(10, 5, 0) + ReLU	Conv (10, 3, 1) + ReLU	Linear(1024) + ReLU	Linear(1024) + ReLU
MaxPool(2)	MaxPool(2)	Linear(512) + ReLU	Linear(1024) + ReLU
Conv(20, 5, 0) + ReLU	Conv (20, 3, 1) + ReLU	Linear(256) + ReLU	Linear(3)
MaxPool(2)	MaxPool(2)	Linear(3)	
Linear(50) + ReLU	Linear(128) + ReLU		
Linear(3)	Linear(3)		

Supplementary Table 1. The architectures of local models for the pneumonia detection task.

Client 1	Client 2	Client 3	Client 4	Client 5	Client 6	Client 7	Client 8	Client 9	Client 10
Conv(10, 5, 0)	Conv(10, 5, 0)	Conv(10, 3, 1)	Conv(10, 3, 1)	Conv(10, 5, 0)	Conv(10, 5, 0)	Linear(1024)	Linear(1024)	Linear(1024)	Linear(1024)
ReLU	ReLU	ReLU	ReLU	ReLU	ReLU	ReLU	ReLU	ReLU	ReLU
MaxPool(2)	MaxPool(2)	MaxPool(2)	MaxPool(2)	MaxPool(2)	MaxPool(2)	Linear(512)	Linear(512)	Linear(1024)	Linear(1024)
Conv(20, 5, 0)	Conv(20, 5, 0)	Conv(20, 3, 1)	Conv(20, 3, 1)	Conv(20, 3, 1)	Conv(20, 3, 1)	ReLU	ReLU	ReLU	ReLU
ReLU	ReLU	ReLU	ReLU	ReLU	ReLU	Linear(256)	Linear(256)	Linear(10)	Linear(10)
MaxPool(2)	MaxPool(2)	MaxPool(2)	MaxPool(2)	MaxPool(2)	MaxPool(2)	ReLU	ReLU		
Linear(50)	Linear(50)	Linear(128)	Linear(128)	Linear(64)	Linear(64)	Linear(10)	Linear(10)		
ReLU	ReLU	ReLU	ReLU	ReLU	ReLU				
Linear(10)	Linear(10)	Linear(10)	Linear(10)	Linear(10)	Linear(10)				

Supplementary Table 2. The architectures of local models for MNIST and Fashion MNIST classification tasks.

Client 1	Client 2	Client 3	Client 4	Client 5	Client 6	Client 7	Client 8	Client 9	Client 10
ResNet*-18	ResNet*-18	ResNet*-18	ResNet*-18	ResNet*-34	ResNet*-34	ResNet*-34	ResNet*-34	ResNet*-50	ResNet*-50
Linear(128)	Linear(128)	Linear(256)	Linear(256)	Linear(128)	Linear(128)	Linear(256)	Linear(256)	Linear(256)	Linear(256)
ReLU	ReLU	ReLU	ReLU	ReLU	ReLU	ReLU	ReLU	ReLU	ReLU
Linear(64)	Linear(64)	Linear(10)	Linear(10)	Linear(64)	Linear(64)	Linear(10)	Linear(10)	Linear(10)	Linear(10)
ReLU	ReLU			ReLU	ReLU				
Linear(10)	Linear(10)			Linear(10)	Linear(10)				

Supplementary Table 3. The architectures of local models for the CIFAR-10 classification task. ResNet* represents the layers of ResNet before the fully connected layer. These ResNet models are pretrained on the ImageNet dataset.

	COVIDx	MNIST	Fashion MNIST	CIFAR-10
Number of local data	$1000 \sim 2000$	5,400	5,400	4,000
Local training batch size	64	64	64	64
Number of proxy data	1,500	6,000	6,000	10,000
Distillation batch size	128	512	512	32

Supplementary Table 4. Summary of datasets.

	Client 1	Client 2	Client 3	Client 4
IndepLearn	91.6 ± 0.3	81.9 ± 0.8	100.0 ± 0.0	100.0 ± 0.0
FedMD	88.4 ± 0.5	84.1 ± 0.7	100.0 ± 0.0	100.0 ± 0.0
FedED	50.0 ± 0.0	50.0 ± 0.0	100.0 ± 0.0	100.0 ± 0.0
DS-FL	77.4 ± 0.5	72.6± 0.6	100.0 ± 0.0	100.0 ± 0.0
FKD	92.3 ± 0.3	74.1 ± 0.9	100.0 ± 0.0	100.0 ± 0.0
PLS	91.7± 0.3	74.9 ± 1.3	100.0 ± 0.0	100.0 ± 0.0
Selective-FD	94.2 ± 0.2	78.7 ± 0.4	100.0 ± 0.0	100.0 ± 0.0

Supplementary Table 5. Local test accuracy on the pneumonia detection task. The knowledge is transferred via soft labels.

MNIST	Fashion MNIST	CIFAR-10
Conv(10,5,0)	Linear(1024)	ResNet*-18
ReLU	ReLU	Linear(128)
MaxPool(2)	Linear(1024)	ReLU
Conv(20,5,0)	ReLU	Linear(64)
ReLU	Linear(10)	ReLU
MaxPool(2)		Linear(10)
Linear(50)		
ReLU		
Linear(10)		

Supplementary Table 6. The architectures of the global model in FedAvg. ResNet* represents the layers of ResNet before the fully connected layer.

	Client 1	Client 2	Client 3	Client 4	Client 5	Client 6	Client 7	Client 8	Client 9	Client 10
Selective-FD	0.79	0.79	0.79	0.79	0.75	0.75	0.75	0.75	0.70	0.70
FedAvg	1.78	1.78	1.78	1.78	0.83	0.83	0.83	0.83	0.70	0.70

Supplementary Table 7. Theoretical computation time (seconds) per communication round in federated training on CIFAR-10.

Communication overhead	Selective-FD			FedAvg	
	Collect proxy samples	Download	Upload	Download	Upload
MNIST	4.7 MB	21.5 KB	20.5 KB	87.4 KB	87.4 KB
Fashion MNIST	4.7 MB	21.5 KB	20.5 KB	7.46 MB	7.46 MB
CIFAR-10	30.7 MB	1.3 KB	1.3 KB	45.1 MB	45.1 MB

Supplementary Table 8. Communication overhead of each client. Our Selective-FD method, while introducing a one-time communication cost for collecting proxy samples, significantly reduces upload and download costs per communication round compared with FedAvg.

Supplementary Figures

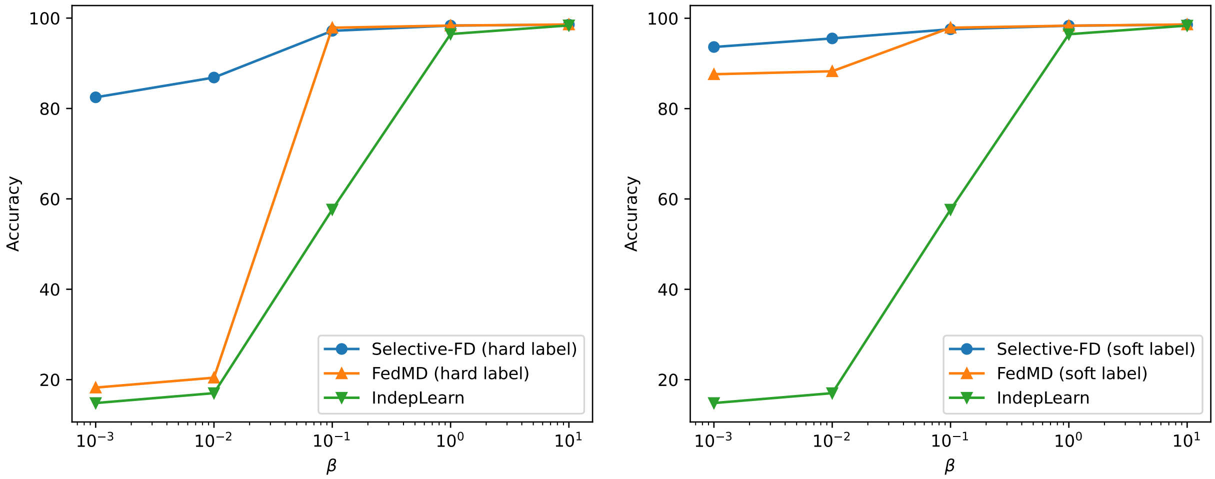

(a) Sharing hard labels (b) Sharing soft labels

Supplementary Figure 1. MNIST classification accuracy in different non-IID settings. When β is set to a smaller value, the data distribution is more non-IID. The knowledge is transferred via (a) hard labels and (b) soft labels.

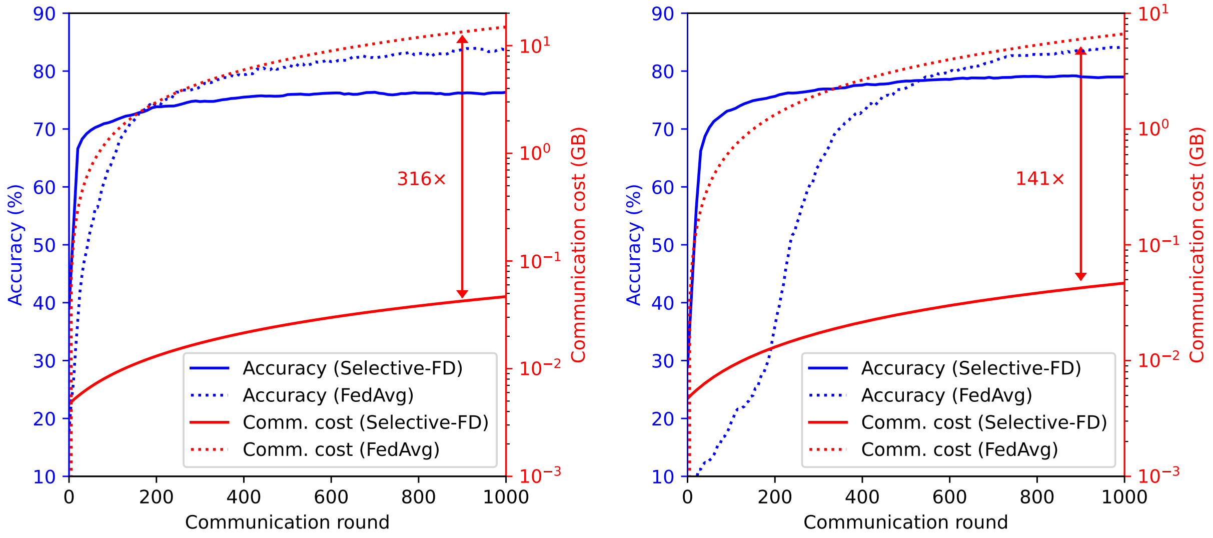

(a) MLP (b) ViT

Supplementary Figure 2. Test accuracy and communication cost as functions of the communication round on the Fashion MNIST classification task. The model architecture is (a) an MLP and (b) a ViT.

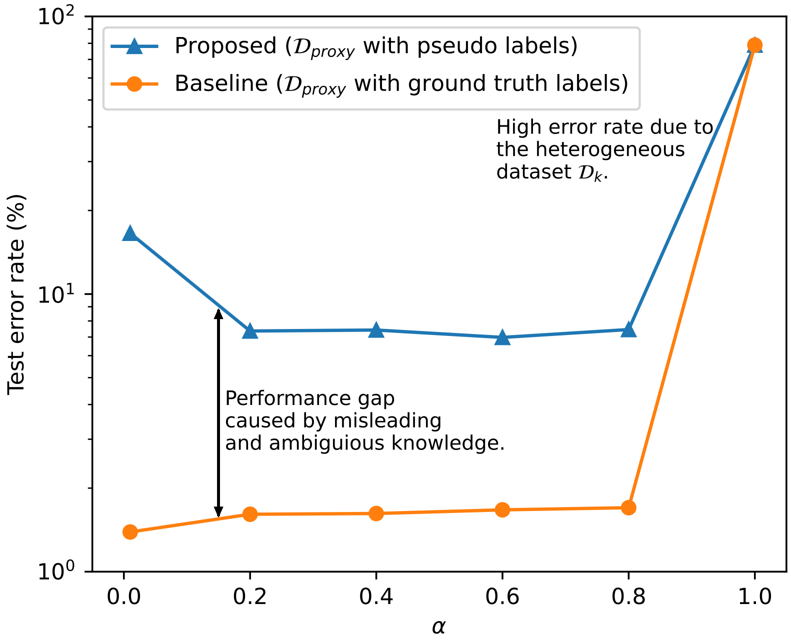

Supplementary Figure 3. Test error rate as a function of α.
