# Supplementary material for: Selective knowledge sharing for privacy-preserving federated distillation without a good teacher
Source: Nat Commun. 2024 Jan 8;15:349. doi: 10.1038/s41467-023-44383-9 (PMC10774276; doi:10.1038/s41467-023-44383-9)
Supplement: Supplementary file 3 — Reporting Summary [file 41467_2023_44383_MOESM3_ESM.pdf]

nature portfolio

Corresponding author(s): Jun Zhang

Last updated by author(s): 14/Nov/23

Reporting Summary

Nature Portfolio wishes to improve the reproducibility of the work that we publish. This form provides structure for consistency and transparency in reporting. For further information on Nature Portfolio policies, see our Editorial Policies and the Editorial Policy Checklist.

Statistics

For all statistical analyses, confirm that the following items are present in the figure legend, table legend, main text, or Methods section.

n/a	Confirmed	
☐	☒	The exact sample size (*n*) for each experimental group/condition, given as a discrete number and unit of measurement
☒	☐	A statement on whether measurements were taken from distinct samples or whether the same sample was measured repeatedly
☒	☐	The statistical test(s) used AND whether they are one- or two-sided<br>*Only common tests should be described solely by name; describe more complex techniques in the Methods section.*
☒	☐	A description of all covariates tested
☒	☐	A description of any assumptions or corrections, such as tests of normality and adjustment for multiple comparisons
☐	☒	A full description of the statistical parameters including central tendency (e.g. means) or other basic estimates (e.g. regression coefficient) AND variation (e.g. standard deviation) or associated estimates of uncertainty (e.g. confidence intervals)
☒	☐	For null hypothesis testing, the test statistic (e.g. *F*, *t*, *r*) with confidence intervals, effect sizes, degrees of freedom and *P* value noted<br>*Give P values as exact values whenever suitable.*
☒	☐	For Bayesian analysis, information on the choice of priors and Markov chain Monte Carlo settings
☒	☐	For hierarchical and complex designs, identification of the appropriate level for tests and full reporting of outcomes
☒	☐	Estimates of effect sizes (e.g. Cohen's *d*, Pearson's *r*), indicating how they were calculated

Our web collection on statistics for biologists contains articles on many of the points above.

Software and code

Policy information about availability of computer code

Data collection	We used publicly available datasets to evaluate the performance of the proposed method. These datasets can be downloaded at https://www.kaggle.com/datasets/andyczhao/covidx-cxr2, http://yann.lecun.com/exdb/mnist/, https://github.com/zalandoresearch/fashion-mnist, and https://www.cs.toronto.edu/~kriz/cifar.html.
Data analysis	We used PyTorch (version 2.0.0) to load the data samples and perform the federated training. Besides, we used Numpy (version 1.21.4) and Matplotlib (version 3.5.0) to analyze the results and draw the figures, respectively. Our code is available at https://github.com/shaojiawei07/Selective-FD.

For manuscripts utilizing custom algorithms or software that are central to the research but not yet described in published literature, software must be made available to editors and reviewers. We strongly encourage code deposition in a community repository (e.g. GitHub). See the Nature Portfolio guidelines for submitting code & software for further information.

Data

Policy information about availability of data

All manuscripts must include a data availability statement. This statement should provide the following information, where applicable:

- Accession codes, unique identifiers, or web links for publicly available datasets
- A description of any restrictions on data availability
- For clinical datasets or third party data, please ensure that the statement adheres to our policy

The datasets used in this paper are publicly available. The chest X-ray images are from the COVIDx dataset, which is available at https://www.kaggle.com/datasets/andyczhao/covidx-cxr2. The benchmark datasets MNIST, Fashion MNIST, and CIFAR-10 are available at http://yann.lecun.com/exdb/mnist/, https://github.com/zalandoresearch/fashion-mnist, and https://www.cs.toronto.edu/~kriz/cifar.html, respectively. The usage of these datasets in this work is permitted under their licenses.

Research involving human participants, their data, or biological material

Policy information about studies with human participants or human data. See also policy information about sex, gender (identity/presentation), and sexual orientation and race, ethnicity and racism.

Reporting on sex and gender	N/A
Reporting on race, ethnicity, or other socially relevant groupings	N/A
Population characteristics	N/A
Recruitment	N/A
Ethics oversight	N/A

Note that full information on the approval of the study protocol must also be provided in the manuscript.

Field-specific reporting

Please select the one below that is the best fit for your research. If you are not sure, read the appropriate sections before making your selection.

☒ Life sciences ☐ Behavioural & social sciences ☐ Ecological, evolutionary & environmental sciences

For a reference copy of the document with all sections, see nature.com/documents/nr-reporting-summary-flat.pdf

Life sciences study design

All studies must disclose on these points even when the disclosure is negative.

Sample size	We evaluated the proposed method on four publicly available datasets, and each dataset contains thousands of samples. Three of them (MNIST, Fashion MNIST, CIFAR-10) are benchmark datasets, which are widely used to compare the performance of different methods. Besides, we also tested our method on the COVIDx dataset. The experimental results show that our method can be implemented in the real-world application.
Data exclusions	No data were excluded from the public datasets.
Replication	We replicated the experiments by using 5 different random seeds. All attempts at replication were successful.
Randomization	The data were randomly divided into training and test sets.
Blinding	The datasets were randomized before getting passed to the training and evaluation. Researchers were blinded to the data partition and sample selection.

Reporting for specific materials, systems and methods

We require information from authors about some types of materials, experimental systems and methods used in many studies. Here, indicate whether each material, system or method listed is relevant to your study. If you are not sure if a list item applies to your research, read the appropriate section before selecting a response.

Materials & experimental systems

n/a	Involved in the study
☒	☐ Antibodies
☒	☐ Eukaryotic cell lines
☒	☐ Palaeontology and archaeology
☒	☐ Animals and other organisms
☒	☐ Clinical data
☒	☐ Dual use research of concern
☒	☐ Plants

Methods

n/a	Involved in the study
☒	☐ ChIP-seq
☒	☐ Flow cytometry
☒	☐ MRI-based neuroimaging
